# Parental Feeding Styles and Their Association With Complementary Feeding Practices and Growth in Mexican Children

**DOI:** 10.3389/fped.2021.786397

**Published:** 2021-12-21

**Authors:** Edith Y. Kim-Herrera, Ivonne Ramírez-Silva, Guadalupe Rodríguez-Oliveros, Eduardo Ortiz-Panozo, Marcela Sánchez-Estrada, Marta Rivera-Pasquel, Rafael Pérez-Escamilla, Juan Angel Rivera-Dommarco

**Affiliations:** ^1^Center for Nutrition and Health Research, National Institute of Public Health, Cuernavaca, Mexico; ^2^Center for Population Health Research, National Institute of Public Health, Cuernavaca, Mexico; ^3^School of Public Health, National Institute of Public Health, Cuernavaca, Mexico; ^4^Department of Social and Behavioral Sciences, Yale School of Public Health, New Haven, CT, United States; ^5^Department of Public Health, National Institute of Public Health, Cuernavaca, Mexico

**Keywords:** infant feeding practices, parental feeding styles, complementary feeding, breastfeeding, growth

## Abstract

**Background:** Complementary feeding practices and corresponding parental feeding styles influence nutritional status in later stages of childhood. Findings on the association of these variables with infant growth remain inconsistent; in Mexico, a research gap exists in this area.

**Research Aims:** (1) To characterize parental feeding styles and complementary feeding practices, and (2) to evaluate the association of parental feeding styles with complementary feeding practices and infant growth at 6 and 9 months of age.

**Methods:** Data were collected from a prospective Mexican birth cohort. Parental feeding styles, complementary feeding practices, and anthropometric data from 263 to 234 mother-child pairs (infants of 6 and 9 months of age, respectively) were analyzed. Logistic and linear regression models were used to determine the associations between variables.

**Results:** The predominant parental feeding style was the “responsive style” (90%). Only 43.7 and 8.1% of 6- and 9-month-old infants, had adequate complementary feeding practices, respectively. At 6 months, mothers who were responsive to satiety signals had 11% lesser possibilities (OR = 0.89, 95% CI [0.80, 0.98]) of their infant having inadequate complementary feeding practices than their counterparts and “pressuring to finish” and “pressuring to eat cereal” sub-constructs were associated with lower weight for length and body mass index Z-scores (*p* = 0.02).

**Conclusions:** A high proportion of infants (>40%) did not meet international recommendations. The “pressuring” parental feeding style sub-constructs were associated with growth indicators in 6-month old infants. This emphasizes the importance of promoting parental responsiveness to infant appetite and satiety signals to achieving adequate complementary feeding practices.

## Introduction

Complementary feeding (CF) is a transitional period in which an infant passes from breastfeeding (BF) to the family diet (solid food). Adequate complementary feeding practices (CFP) during the first 2 years of life are key to proper infant growth, nutrition, and development. During the first year of life, BF and CF coincide with a period of behavioral modeling which determine long-term eating habits, growth, and development outcomes, as well as future metabolic responses linked to non-communicable diseases like type 2 diabetes (1).

In 2019, globally around 44% of infants between zero and 5 months of age were exclusively breastfed, 71% of infants between 6 and 8 months received CF, and 28% met food diversity recommendations (2). In Mexico, 28.6% of infants under 6-month-old received exclusive BF, 91.2% of infants between 6 and 9 months received CF, and 70.9% met food diversity recommendations (3).

The interruption of exclusive BF coincides with an early introduction of CF and of ultra-processed foods, including infant formulas and sugar-sweetened beverages (SSB) (4). In Mexico, 35% of infants between 6 and 11.9 months consume SSB and ~20% consume desserts and unhealthy snacks (5). In recent years, the availability and consumption of energy-dense foods and SSB has risen among children in low- and middle-income countries. Consumption of these products can affect the health and nutrition of children by displacing nutrients and leading to inadequate dietary intake (6). Studies in animal and human models have shown that introduction of CF before 4 months of age and high protein intake, were associated with greater weight gain and obesity during childhood (7) which may lead to increased risk of cardiovascular disease in later stages of life (8).

During the CF period, the age of food introduction, genetic predisposition, and parental feeding styles (PFS), determine both food preferences and consumption patterns which may influence dietary habits throughout life (9). PFS, the attitudes that characterize parental actions to maintain or modify child eating behaviors, are based on degree of parental control and responsiveness shown during child feeding (10). These styles influence CFP by establishing the quantity, quality, and frequency of foods offered to infants. Controlling PFS (i.e., the restrictive or pressuring style), can lead to poor infant self-regulation of intake by overriding appetite and satiety mechanisms that can lead to an increased risk of overweight and obesity across the life cycle (11). Responsive PFS, in which the caregiver adequately interprets the appetite and satiety signals of the infant, can lead to healthy eating habits and promote infant growth (12).

Empirical findings on the association of PFS with CFP and growth remain inconsistent, and in Mexico a notable research gap exists in this area (13). Therefore, this study aims to characterize PFS and CFP, and to evaluate the association of PFS with CFP and infant growth at 6 and 9 months of age.

## Materials and Methods

### Design

Data was used from an open ongoing prospective cohort study of mother-child pairs called *MAS-Lactancia*, whose overall goal is to examine appetite and satiety self-regulation as a mediator of maternal and infant health outcomes. All study procedures were approved by the Research, Biosafety, and Ethics Committees of the National Institute of Public Health of Mexico (CI-1281-2016).

### Sample

**Figure 1:**
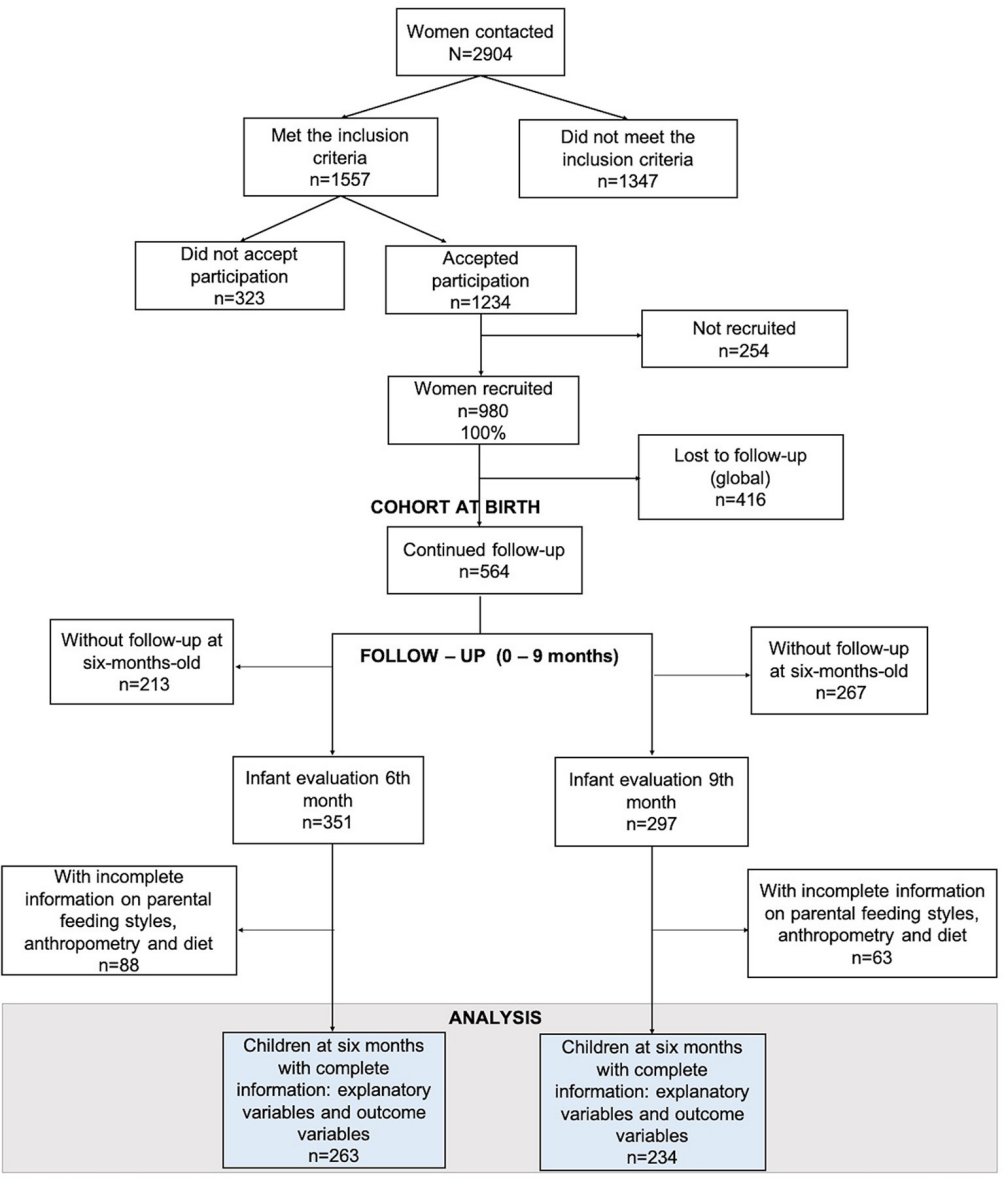
Study sample from the *MAS-Lactancia* birth cohort.

A total of 2,874 women attending to a federal government healthcare facility (HF) were screened based on: having no personal history of high blood pressure, hypertensive diseases of pregnancy, endocrine disorders, and diseases of the kidney, liver, heart, or vascular system (Figure 1). Mothers were residents of Cuernavaca city; Mexico. Infants with intrauterine growth restriction (or low weight-for-gestational-age), or conditions that affected appetite, food intake, or growth (e.g., congenital diseases, epilepsy, cleft palate, and food allergies), were excluded from the study as well as infants with malformations that prevented accurate anthropometric measurements (14). Of the 2,874 women screened, the *MAS-Lactancia* cohort recruited 980 women between 18–39 years old at the 16–20 week stage of a singleton pregnancy. Of these women, 42.4% were lost to follow-up due to work or school responsibilities, death of the infant, or because the mother could not be located despite repeated telephone calls and messages, as well as home visits. All the participants received personalized BF counseling from recruitment to 18 months of infant life in order to promote adequate CFP.

At 6 and at 9 months, respectively, 88 and 63 infants with incomplete information regarding CFP, PFS, and anthropometric measurements were excluded from the analysis. Eventually, this study analyzes and presents the findings of 263 and 234 participants at 6 and 9 months, respectively (Figure 1).

### Measurement

#### Exposure Variables

##### Parental Infant Feeding Styles

Data analyzed included three PFS and their respective sub-constructs of the Infant Feeding Style Questionnaire (IFSQ): (1) Pressuring style: pressuring to finish, pressuring to eat cereal, and pressuring to soothe, (2) Restrictive style: restrictive in dietary quantity, and quality, and (3) Responsive style: responsive in attention and responsive to satiety. These PFS were selected based on their previous association with diet and weight in children (12), and adequate model fit (15). The “indulgence” and “laissez-faire” PFS were not used in this study due to limited information, reliability on their use within Latino population, and lack of an adequate model fit (15).

A 5-point Likert scale was used to assess response options of the IFSQ and scored PFS and their sub-constructs (1 = never, 2 = rarely, 3 = half the time, 4 = most of the time, 5 = always). An additive score was calculated for each PFS and corresponding sub-constructs, and used as a continuous variable. Higher scores were interpreted as having greater affinity with the corresponding PFS or sub-construct (16).

#### Outcome Variables

##### Complementary Feeding Practices at 6 and 9 Months of Age

We classified CFP according to two components: BF and CF. The BF component was further classified into two categories (adequate or inadequate, according to international recommendations) according to the infant's age and type of BF received (Supplementary Table 1). The CF component was evaluated with four indicators based on the WHO recommendations for infant feeding: (1) age at first introduction of solid foods (adequate or inadequate), (2) minimum food diversity (yes or no), (3) consumption of SSB (yes or no), and (4) consumption of ultra-processed foods (yes or no) (4). Then, three categories of CFP based on BF and CF practices were defined in accordance with international recommendations: adequate, moderately adequate, and inadequate (Supplementary Table 1).

##### Infant Growth

Weight-for-length (W/L) and body mass index (BMI) Z-scores at 6 and 9 months of age were obtained with the statistical software STATA^®^ version 14.0 (StataCorp, TX, USA, 2015), according to WHO recommendations. Abdominal circumference was measured in centimeters.

#### Covariates

We considered for inclusion in multivariate analyses: (1) infant characteristics: weight at birth in grams, morbidity (e.g., acute respiratory infections and gastrointestinal infections, and/or hospitalizations history before follow-up), and type of primary caregiver (e.g., parents, grandmother, aunt, babysitter/teacher, nursery staff), and (2) mother and household characteristics: number of children, formal education (years), and BMI at 16–20 weeks of gestation. A Household Wealth Index (HWI) was generated using principal component analysis, which included housing conditions (housing type, floor, walls, and roofing construction materials), water and sanitation services, ownership of home appliances, electronics, and number of rooms (17). The first component explained 30% of the variability, which was interpreted as a proxy of socioeconomic index, divided into tertiles, the lowest reflecting the poorest conditions.

#### Data Collection

From March 2016 to December 2019, study staff invited women to participate during prenatal care sessions at HF. Written informed consent was obtained for all women who chose to participate in the study. PFS data was collected with the IFSQ, previously validated in a Latino population (15) and adapted to the Mexican population. The IFSQ was applied to mothers and main caregivers of infants with 6 and 9 months of age, and used a self-report format (Supplementary Table 2).

Information on feeding practices was obtained through three methodologies. The first two, *status-quo* and recall as recommended by the World Health Organization (WHO) (18), were supplemented with a 24-h recall questionnaire of multiple-iterative steps, to characterize infant food intake with greater precision (14).

Anthropometric measurements were taken (19) by trained and standardized personnel (20). Training and standardization were carried out before data collection and staff re-standardizations were performed every 6 months. To measure infant weight, a pediatric scale (model Tanita^®^ 1584 Baby Scale) with an accuracy of 10 g was used. Scales were calibrated daily with a known reference weight, and all measurements were taken in duplicate. Length was measured using a wooden stadiometer with an accuracy of 1 mm (Schorr). Abdominal circumference was measured with a Lufkin^®^ model W606PM tape.

Covariate data was obtained from the recruitment and screening questionnaires, infant caregiver questionnaire, morbidity questionnaire, and socioeconomic and demographic characteristics questionnaire. The interviewers were previously trained and standardized to apply all the questionnaires.

#### Data Analysis

Means and standard deviations were obtained for continuous variables, and percentages for categorical variables. Student *t*-tests, chi-square, and Fisher's exact tests were performed to estimate differences between participants and between the baseline characteristics of study mothers. Also, we compared the distributions of CFP by parental feeding style at 6- and 9-months using Fisher's exact tests.

To evaluate the association between CFP and PFS, multinomial logistic regression models were performed using adequate feeding practices as the reference category. Relative risk ratios and 95% confidence intervals (CI) were interpreted as the odds ratio (OR). To evaluate the association between PFS and growth, multiple linear regression models were used, where *β* coefficients and 95% CIs were obtained. Regression analyses were performed for each PFS sub-construct and were adjusted for covariates selected *a priori* based on the directed acyclic graph methodology. A supplementary analysis was also performed to compare age at CF introduction and formula consumption category. In addition, we replicated our main multinomial regression analysis using repeated measures from both 6 and 9 months, defining the exposure as increased, decreased, or without change in PFS sub-construct scores. We also replicated our main linear regression analysis, using as outcome the difference of W/L between 9 and 6 months. All analyses were conducted with STATA 14.0 (StataCorp, TX, USA, 2015).

## Results

We analyzed a subset of 263 and 234 mother-child pairs at 6 and 9 months, respectively, with complete information on PFS, anthropometry, and diet. A total of 564 participants were lost to follow-up; however, there were no differences in sociodemographic characteristics between the study and the sample subset excluded from analysis, at 6 and 9 months (*p* > 0.05), except for the mother's age (*p* = 0.01; Supplementary Table 3).

### Participant Characteristics

Infant characteristics, PFS, and PFS sub-constructs are shown in Table 1. Mean birthweight of the infants was 3.1 (*SD* = 0.4) kilograms. Mean W/L Z-scores at 6 and 9 months were 0.11 (*SD* = 1.0) and −0.03 (*SD* = 1.0), respectively. At both 6 and 9 months, the “responsive” PFS scored the highest (mean 4.1, *SD* = 0.5; and 4.2, *SD* = 0.4, respectively), as well as the “responsive to satiety” sub-construct (mean 4.4, *SD* = 0.4, at each follow-up event) and the “responsive in attention” sub-construct (mean 3.6, *SD* = 1.0, at 6 months and 3.8, *SD* = 0.9 at 9 months). More than 50% of the infants at each follow-up event were females and had a history of illness or hospitalization prior to the staff visits (morbidity). The parents were the main caregivers of the infants (86.3 and 84.2% at 6 and at 9 months, respectively). Other caregivers were grandparents, uncles, teachers or neighbors (information not shown).

**Table 1:** Descriptive characteristics and parental feeding styles of the study sample^a^.

**Infant characteristics**	**6 months**		**9 months**	
	***n***	**M (SD)**	***n***	**M (SD)**
Age (months)	263	6.5 (0.4)	234	9.3 (0.5)
Birthweight (kg)	259	3.1 (0.4)	230	3.1 (0.4)
**Weight (kg)**				
Females	136	7.2 (0.8)	121	7.90 (0.9)
Males	127	7.6 (0.8)	113	8.41 (0.9)
**Length (cm)**				
Females	136	65.0 (2.0)	121	68.5 (2.3)
Males	127	66.2 (2.0)	113	70.0 (2.2)
Weight-for-length z-score^b^	263	0.1 (1.0)	234	−0.0 (1.0)
BMI for age z-score^b^	263	0.0 (1.0)	234	−0.0 (1.0)
Abdominal circumference (cm)	236	42.4 (2.4)	156	43.1 (3.3)
**Parental feeding styles scores^c^**				
Restrictive	263	2.9 (0.4)	234	2.9 (0.5)
Pressuring	263	3.1 (0.5)	234	3.0 (0.5)
Responsive	263	4.1 (0.5)	234	4.2 (0.4)
**Parental feeding style sub-construct scores**				
Restrictive in quantity	263	3.5 (0.8)	234	2.9 (0.8)
Restrictive in quality	263	2.4 (0.5)	234	1.9 (0.5)
Pressuring to finish	263	3.1 (0.7)	234	3.2 (0.8)
Pressuring to eat cereal	263	2.9 (0.6)	234	3.0 (0.6)
Pressuring to soothe	263	3.0 (0.8)	234	2.9 (0.8)
Responsive to satiety	263	4.4 (0.4)	234	4.4 (0.4)
Responsive in attention	263	3.6 (1.0)	234	3.8 (0.9)

*BMI, body mass index*.

a *MAS-Lactancia cohort 2016–2018, differences in the number of observations per contact and covariates*.

b *Weight-for-length and BMI indicators according to WHO growth standards*.

c *Parental feeding styles assessed by the Infant Feeding Style Questionnaire*.

On average, mothers were 27.1 (*SD* = 5.1) years old, with 13.0 (*SD* = 3.2) years of formal education, and the mean BMI at recruitment was 26.1 (*SD* = 4.0). More than 50% of the mothers had a partner, were first-time mothers, and were employed. Around 30% of mothers were in the highest tertile of the HWI, and 34% were in the lowest tertile of the HWI (information not shown).

### Infant Feeding Practices

Adequacy of CFP by BF and CF at 6 and 9 months of age is shown in Table 2. At 6 and 9 months, 43.7 and 8.1% of infants, respectively, had adequate CFP. Infants older than 6 months received mixed BF both with and without infant formula and BF was adequate in 87.1% of infants at 6 months and 76.5% of infants at 9 months. The mean age of CF introduction was 4.3 months (*SD* = 2.2), and occurred on average 2.6 months earlier for those consuming formula, compared to those who were still receiving BF (Supplementary Table 4). The mean age of CF introduction on infants with adequate CFP was higher (*p* = 0.012) compared to those with inadequate CFP (mean 5.9, *SD* = 0.4 and 2.4, *SD* = 2.1, respectively). Approximately 50% of infants had inadequate food diversity (<4 food groups in formula-fed infants) at 9 months of age. Nearly one of every three infants and one of every two infants at 6 and at 9 months of age, respectively, consumed ultra-processed foods. Around one of every 10 infants at 6 months of age, and one of every three infants at 9 months, consumed SSB. Nearly one of every three infants at 6 months of age, and one of every two infants at 9 months, consumed both ultra-processed foods and SSB (Table 2).

**Table 2:** Infant feeding practices by breastfeeding and complementary feeding components, at 6 and 9 months of infant age.

**Infant feeding practices**	**6 months (*****n*** **=** **263)**					**9 months (*****n*** **=** **234)**				
	**Global**	**Adequate**	**Moderately adequate**	**Inadequate**	***p*-value^‡^**	**Global**	**Adequate**	**Moderately adequate**	**Inadequate**	***p*-value^‡^**
	**%**	**%**	**%**	**%**		**%**	**%**	**%**	**%**	
Complementary feeding practices (global)	100.0	43.7	11.8	44.5		100.0	8.1	40.6	51.3	
**Type of breastfeeding^a^**										
Mixed (BF y CF)	42.2	67.6	18.9	13.5	**<0.01**	41.0	10.4	50.0	39.6	**<0.01**
Mixed (BF, F y CF)	44.9	33.9	8.5	57.6		35.5	10.8	56.6	32.5	
No breastfeeding (F y CF)	12.9	0.0	0.0	100.0		23.5	0.0	0.0	100.0	
**Assessment of BF^b^**										
Adequate	87.1	50.2	13.5	36.2	**<0.01**	76.5	10.6	53.1	36.3	**<0.01**
Inadequate	12.9	0.0	0.0	100.0		23.5	0.0	0.0	100.0	
**Food diversity**										
Yes	14.8	23.1	28.2	48.7	**<0.01**	48.7	16.7	15.8	67.5	**<0.01**
No	85.2	47.3	8.9	43.8		51.3	0.0	64.2	35.8	
**SSB consumption**										
Yes	12.2	0.0	0.0	100.0	**<0.01**	35.1	0.0	0.0	100.0	**<0.01**
No	87.8	49.8	13.4	36.8		65.0	12.5	62.5	25.0	
**UP food consumption**										
Yes	29.3	0.0	40.3	59.7	**<0.01**	53.8	0.0	29.4	70.6	**<0.01**
No	70.7	61.8	0.0	38.2		46.2	17.6	53.7	28.7	
UP+SSB consumption	31.9	0.0	36.9	63.1	**<0.01**	58.6	0.0	27.0	73.0	**<0.01**
**Age at introduction of CF**										
Adequate	66.9	65.3	17.6	17.1	**<0.01**	–	**–**	**–**	**–**	**–**
Inadequate	33.1	0.0	0.0	100.0		**–**	**–**	**–**	**–**	**–**
Mean (SD) years	4.3 (2.2)	5.9 (0.4)	5.6 (0.7)	2.4 (2.1)	**0.012**	**–**	**–**	**–**	**–**	**–**

*p-values are bolded p < 0.05. SSB, sugar-sweetened beverages; UP, ultra-processed foods; BF, breastfeeding; CF, complementary feeding; F, infant formula*.

a *Type of breastfeeding based on WHO classification*.

b *Breastfeeding assessment according to infant age*.

‡ *Fisher exact test*.

### Parental Feeding Styles and Complementary Feeding Practices

The association between PFS and CFP is shown in Table 3. Mothers who were responsive to signals of the “satiety” sub-construct had an 11% lower possibility (*p* = 0.02) of their infant having inadequate CFP at 6 months of age than their counterparts. There were no significant differences (*p* > 0.05) for the others sub-constructs.

**Table 3:** Association between parental feeding style sub-constructs and complementary feeding practices.

	**6 months (*****n*** **=** **263)**						**9 months (*****n*** **=** **234)**					
	**Complementary feeding practices**						**Complementary feeding practices**					
	**Moderately adequate^†^**			**Inadequate^†^**			**Moderately adequate^†^**			**Inadequate^†^**		
**Parental feeding style sub-constructs**	**OR**	**CI 95% (LL, UL)**	***p-*value**	**OR**	**CI 95% (LL, UL)**	***p-*value**	**OR**	**CI 95% (LL, UL)**	***p-*value**	**OR**	**CI 95% (LL, UL)**	***p-*value**
Pressuring to finish	0.98	0.90, 1.07	0.65	1.03	0.98, 1.10	0.26	1.00	0.86, 1.16	1.00	1.02	0.88, 1.18	0.81
Pressuring to eat cereal	1.04	0.86, 1.27	0.66	0.92	0.81, 1.04	0.19	0.85	0.60, 1.21	0.37	0.87	0.61, 1.23	0.43
Pressuring to soothe	0.92	0.79, 1.07	0.29	1.04	0.94, 1.14	0.44	0.97	0.73, 1.30	0.87	1.02	0.77, 1.37	0.86
Restrictive in quality	1.06	0.89, 1.25	0.52	1.05	0.94, 1.17	0.40	1.00	0.74, 1.34	0.99	0.97	0.72, 1.31	0.85
Restrictive in quantity	0.92	0.80, 1.07	0.28	1.01	0.91, 1.13	0.82	0.94	0.79, 1.12	0.49	0.91	0.77, 1.09	0.31
Responsive to satiety	1.04	0.88, 1.24	0.61	0.89	0.80, 0.98	**0.02**	0.82	0.57, 1.17	0.27	0.89	0.63, 1.28	0.54
Responsive in attention	0.98	0.87, 1.09	0.68	1.01	0.94, 1.09	0.73	0.90	0.70, 1.15	0.40	0.93	0.72, 1.19	0.56

*Assessment of the seven parental feeding style sub-constructs corresponding to pressuring, restrictive and responsive feeding styles. p-values are bolded if p < 0.05*.

† *Versus category of reference of adequate complementary feeding practices*.

*OR, odds ratio; CI, confidence intervals; LL, lower limit; UL, upper limit. Logistic regression models adjusted by morbidity, birthweight, type of caregiver, breastfeeding, educational background, number of children, BMI at recruitment, and mother's Household Wealth Index (HWI) tertile*.

### Parental Feeding Styles and Growth

Highest scores from sub-constructs “pressuring to finish” and “pressuring to eat cereal” were associated with infants at 6 months of age with lower W/L and BMI Z-scores (*p* ≤ 0.02), and were marginally associated with a lesser abdominal circumference (*p* = 0.05). Likewise, the “responsive to satiety” sub-construct was marginally associated with lesser abdominal circumference (*p* = 0.09). At 9 months of age, a marginal association was present between “pressuring to finish” and “pressuring to eat cereal” sub-constructs, with lower W/L Z-score and BMI Z score (*p* = 0.06 and *p* = 0.07, respectively). No differences were identified from others sub-constructs (Table 4).

**Table 4:** Association between parental feeding style sub-construct scales and growth indicators.

**Sub-construct scales^a^ Growth indicators^b^**	**6 months**				**9 months**			
	***n***	**β**	**CI 95%LL, UL**	***p-*value**	***n***	**β**	**CI 95%LL, UL**	***p-*value**
**Pressuring to finish (score)**								
Weight-for-length z-score	238	−0.03	−0.05, 0.00	**0.02**	214	−0.02	−0.04, 0.00	0.06
Body mass index z-score	238	−0.03	−0.05, 0.00	**0.02**	214	−0.02	−0.05, 0.00	0.07
Abdominal circumference (mm)	236	−0.06	−0.13, 0.00	0.05	211	−0.04	−0.12, 0.04	0.34
**Pressuring to eat cereal (score)**								
Weight-for-length z-score	238	−0.07	−0.13, −0.02	**0.01**	214	0.00	−0.05, 0.05	0.92
Body mass index z-score	238	−0.07	−0.12, −0.02	**0.01**	214	0.00	−0.06, 0.05	0.91
Abdominal circumference (mm)	236	−0.20	−0.34, −0.02	**0.01**	211	−0.01	−0.19, 0.16	0.88
**Pressuring to soothe (score)**								
Weight-for-length z-score	238	−0.03	−0.07, 0.01	0.15	214	−0.01	−0.05, 0.03	0.67
Body mass index z-score	238	−0.03	−0.07, 0.01	0.21	214	−0.01	−0.06, 0.03	0.63
Abdominal circumference (mm)	236	−0.02	−0.13, 0.09	0.75	211	0.06	−0.08, 0.21	0.40
**Restrictive in quality (score)**								
Weight-for-length z-score	238	0.01	−0.04, 0.05	0.82	214	−0.01	−0.06, 0.04	0.64
Body mass index z-score	238	0.00	−0.04, 0.05	0.89	214	−0.01	−0.06, 0.04	0.61
Abdominal circumference (mm)	236	−0.01	−0.13, 0.11	0.88	211	0.05	−0.12, 0.22	0.54
**Restrictive in quantity (score)**								
Weight-for-length z-score	238	−0.02	−0.06, 0.02	0.39	214	−0.01	−0.05, 0.03	0.49
Body mass index z-score	238	−0.02	−0.05, 0.02	0.43	214	−0.01	−0.05, 0.03	0.53
Abdominal circumference (mm)	236	0.03	−0.07, 0.13	0.55	211	−0.02	−0.16, 0.11	0.77
**Responsive to satiety (score)**								
Weight-for-length z-score	238	0.01	−0.03, 0.05	0.66	214	0.00	−0.05, 0.04	0.86
Body mass index z-score	238	−0.01	−0.05, 0.02	0.43	214	0.00	−0.03, 0.04	0.89
Abdominal circumference (mm)	236	−0.08	−0.16, 0.01	0.09	211	−0.05	−0.17, 0.07	0.42
**Responsive in attention (score)**								
Weight-for-length z-score	238	−0.01	−0.04, 0.02	0.44	214	0.00	−0.03, 0.04	0.96
Body mass index z-score	238	0.01	−0.03, 0.05	0.63	214	0.00	−0.05, 0.04	0.84
Abdominal circumference (mm)	236	0.02	−0.09, 0.13	0.73	211	−0.04	−0.20, 0.12	0.64

*p-values are bolded if significant a p < 0.05*.

a *Assessment of the seven parental feeding style subscales corresponding to pressuring, restrictive, and responsive feeding styles*.

b *Lineal regression models adjusted for morbidity, birthweight, type of caregiver, breastfeeding, educational background, number of children, BMI at recruitment, and mother's Household Wealth Index (HWI) tertile*.

*CI, confidence intervals; LL, lower limit; UL, upper limit*.

## Discussion

Our results show that the “responsive” style was the predominant PFS for Mexican mothers. Nevertheless, more than 40% of the infants evaluated had a suboptimal CFP and not compliant with international feeding recommendations. Also, infants had inadequate food diversity and already consumed ultra-processed foods and SSB as a part of their diets. Likewise, between 87.1 and 76.5% of infants evaluated (at 6 and 9 months, respectively) received age-appropriate BF.

Mothers of 6-month-old infants who were “responsive to satiety” signals had lower possibilities of their infants having inadequate CFP than their counterparts; in contrast, the “pressuring to finish” and “pressuring to eat cereal” sub-constructs were associated with lower W/L and BMI Z-scores, as well as lesser abdominal circumference, among 6-month-old infants. This difference was statistically not significant (*p* < 0.05) at 9 months of age, possibly due to the reduction of the study sample at follow-up. However, the results presented the same direction compared to those obtained at 6 months.

Related to the existing literature, there are no recent studies and data on PFS in Mexican mothers of children under 1 year of age. However, PFS have been explored in low income Hispanic mothers of preschool-age children (10) and in low income African-American and Latino mothers of infants (15, 21), both groups residing in the United States. Additionally, PFS have been studied in Latin America but focused on preschool-age children (13). Also the results in Latino and African-American mothers showed that the “responsive style” was the predominant PFS. However, in our sample at 9 months, the second predominant PFS was “pressuring style.” This result highlights the importance of early identification of controlling eating behaviors in parents, since it could cause poor regulation of infant intake, and affect weight gain during childhood.

In our sample, CF was introduced earlier than is recommended, particularly in formula-fed infants. Similar results were found in a birth cohort from Brazil (1) and in a randomized controlled trial in the Netherlands (22), where semi-solid and liquids different from BF were introduced before 6-months of age. According to Schneider et al., infants who introduced CF before 4 months of age were found to have a higher risk of childhood overweight (1). The early initiation of CF displaces breast milk consumption with other foods or liquids (e.g., processed juices, SSB, or non-human milk), which poses a nutritional and health risk for infants (7, 12).

Results from the National Health and Nutrition Survey 2018–2019 (ENSANUT by its acronym in Spanish) showed that food diversity is around 70.9% in Mexican infants between 6 and 23 months-old (23). In our study sample <50% of 6- and 9-month-old infants comply with this indicator. Additionally we found, consistent with previous research on Mexican infants (5), that from 6-month of age, infants were already consuming SSB and ultra-processed foods, and continued this practice as they grew older. Consumption of SSB and ultra-processed foods at early age represent a major public health concern, due to the low nutritional quality of these products and their potential of altering the regulatory mechanisms of appetite and satiety (6, 8). Sound infant feeding policies (i.e., those currently in place in Mexico), must be implemented to prevent consumption of processed and ultra-processed foods, increase food diversity, and avoid non-nutritional diets in young children (3, 5).

According to our results, between 76.5 and 87.1% of infants received age-appropriate BF; this proportion was higher than previously reported in Mexican infants (40.2% for infants between 6 and 11.9 months-old) (5). These relative high rates of BF may be attributed to counseling on infant feeding, and particularly on BF, provided to mothers at follow-up. Individual counseling may increase parental knowledge, confidence and self-efficacy, promoting positive feeding behaviors like BF (24).

Recent data from ENSANUT 2018–2019 in Mexico showed that infant under 12 months of age had a consumption of infant formula around 43% (23). Our results are comparable with these national data since around 45% of 6-month-old infants and 35% of 9-month-old infants reported consuming infant formula in addition to BF.

In accordance with Thompson et al., we found that the “responsive” feeding style and specifically, the “responsive to satiety” sub-construct was associated with lower odds of inadequate CFP at 6 months of age (21). “Responsive” feeding styles has been associated with healthy eating patterns and with a lower risk of obesity (16). Promoting the “responsive feeding” style is particularly important during early infancy to achieve an adequate transition to CF, since at around 6 months of age infants should start the process of incorporating healthy new foods into the diet to develop healthy eating habits (12, 25). Also, like previously reported, it is plausible that the study mothers who practiced BF (75%), would have supportive infant feeding attitudes and beliefs, and better identify their infants' signs of appetite; thereby, favoring positive CFP (1, 16). However, at 9 months, no association was observed between categories of CFP and parental feeding style sub-constructs (*p* > 0.05). One possible explanation is that unlike the 9-month-old infants are already immerse in the family diet, infant around 6 months are going through the period of transition to family diet and we speculate that mothers could be more responsive and careful of infant feeding which allow us to identify associations between “responsive” sub-construct and CFP. Another possible explanation of the lack of associations at 9 months is that the maternal behavior with regard to infant feeding could variate according to specific feeding situations like refusal to eat some kind of food. It is possible that as the infant grows more controlling practices appear, creating a unfavorable feeding environment (12).

In relation to W/L indicator, similar results were presented in a systematic review, where the authors found that “pressuring style” is related with lower weight gain or weight status on infants (26). Another finding was that mothers are concerned about their infant's size, therefore they pressure their children to eat (10, 26). Also our findings are similar with a previous cross-sectional study that reported an association between the “pressuring” feeding style and lower W/L Z-scores (9). Furthermore, Milanaik et al. found that mothers of under 9-month-old infants were more likely to add cereal to the infant's bottle, believing that this practice would improve growth and sleep patterns (27). Another study found that Mexican mothers offered cereal to young children to promote growth, development, and wellbeing, as considering it healthy, nutritious, practical, and easy to prepare (28).

“Pressuring” during childhood may mark the beginning of overfeeding behaviors in children and overweight in later stages of life. These findings highlight the importance of discouraging the “pressuring” feeding style to prevent childhood obesity, and the need for continued research in PFS and child growth.

### Limitations

Some limitations of the present study should be considered. Due to the lack of variability in some PFS (90% of mothers practiced the “responsive” style), it was not possible to describe the characteristics of CFP and growth through each PFS. Additionally, the potential for residual confusion cannot be ruled out, given that there were unmeasured variables (i.e., paternal role and social desirability bias, as mothers may have responded according to expected standards). We present here cross-sectional statistical analyses at 6 and 9 months, although a longitudinal analysis was also performed to evaluate the 6 to 9-month period which identified the expected direction of results (similar to the findings of the cross-sectional study), however, the associations were not statistically significant (Supplementary Table 5), possibly due to the short study period, low variability between outcome variables within these periods, or the reduction of the study sample. For this reason, the main findings of this study are derived in the cross-sectional analysis with the aim to contribute evidence around the association between PFS, and CFP and infant growth across different phases of early childhood.

Some strengths of our study should also be considered. To our knowledge, this study is among the first to examine the association of PFS with CFP and growth among Mexican infants below 1 year of age. In Mexico, evidence around factors which influence CFP is scarce, and to our knowledge there are limited studies focused on PFS. Additionally, our data come from the “*MAS-Lactancia*” study, a current open prospective birth cohort, relevant in the present epidemiological and nutritional context in Mexico. Likewise, the methodology used in the study was designed to collect detailed information on feeding practices, PFS, and growth indicators during infancy which allowed to provide evidence around the characterization of CFP and PFS. Another strength of the present study is that we assessed PFS with the IFSQ, which has been previously validated in Latino populations (15) and adapted to the Mexican population (14).

## Conclusions

The results of this study show a high proportion of infants with suboptimal CFP, and that some PFS sub-constructs (i.e., “responsive to satiety”) were associated with adequate CFP only at 6 months, while some others (i.e., “pressuring to finish”) were negatively associated with anthropometric indicators of growth and adiposity. Our findings suggest that promoting “responsive” PFS and identifying signals of appetite and satiety in early childhood may have a positive impact in CFP at 6 months of age. Further research is needed to better understand the impact of PFS on infant diet, growth, and development in Mexico and beyond. Nonetheless, our findings can be used to improve infant feeding guidelines and policies and can be used to improve current early dietary counseling to parents of toddlers focusing on responsive feeding that sensitize parents to infant's cues during feeding to improve infant and complementary feeding practices, promote a self-regulation of energy intake and a healthy weight and prevent overweight and obesity on childhood.

## Data Availability Statement

The datasets presented in this article are not readily available because the data comes from an ongoing prospective cohort that continues to be updated. Requests to access the datasets should be directed to the corresponding author IR-S.

## Ethics Statement

The studies involving human participants were reviewed and approved by Comité de Ética en Investigación. Instituto Nacional de Salud Pública. Registro ante CONBIOÉTICA: 17CEI00120130424 Registro ante COFEPRIS: 13 CEI 17 007 36 FWA: 00015605. Written informed consent to participate in this study was provided by the participants' legal guardian/next of kin.

## Author Contributions

EK-H performed the formal analysis and wrote the paper. JR-D and IR-S conceptualized the study. IR-S and EO-P performed and reviewed the statistical analysis. EK-H and IR-S wrote the initial drafts. GR-O, MS-E, MR-P, RP-E, and JR-D provided substantive inputs, which were incorporated in the final draft. All authors had the responsibility for final content, and read and approved the final manuscript.

## Funding

This study was provided by the National Council of Science and Technology (for its Acronym in Spanish CONACYT) CONACYT 0233439, 290275, and 2574562 and Fundación Gonzalo Rio Arronte (3139). The funding agencies had no role in the study design, collection, analysis or interpretation of the data, writing the manuscript, or the decision to submit the paper for publication.

## Conflict of Interest

The authors declare that the research was conducted in the absence of any commercial or financial relationships that could be construed as a potential conflict of interest. The handling editor declared a past co-authorship with one of the author JR-D.

## Publisher's Note

All claims expressed in this article are solely those of the authors and do not necessarily represent those of their affiliated organizations, or those of the publisher, the editors and the reviewers. Any product that may be evaluated in this article, or claim that may be made by its manufacturer, is not guaranteed or endorsed by the publisher.
