# Supplementary material for: Parental Feeding Styles and Their Association With Complementary Feeding Practices and Growth in Mexican Children
Source: Front Pediatr. 2021 Dec 21;9:786397. doi: 10.3389/fped.2021.786397 (PMC8724423; doi:10.3389/fped.2021.786397)
Supplement: Supplementary file 1 [file Data_Sheet_1.pdf]

SUPPLEMENTARY TABLES

Supplementary Table 1.

Classification and Components of Infant Feeding Practices in Mexican Infants of the MAS-Lactancia Cohort (2016-2018)

Component I. BF	Definition / criteria
Exclusive breastfeeding	Only breast milk without other liquids or food. Whey, medicines, vitamins, and minerals are allowed.<br>Adequate: up to six months.
Partial breastfeeding	Formula, other milks, and breast milk (without food).<br>Adequate: from six months of age, but not older than 6.5 months
Mixed breastfeeding	Breast milk and food (including other milks).<br>Adequate: From six months of age.
No breastfeeding	Not having reported consuming breast milk<br>Inadequate for any age group.
**Component II. CF**	**Definition / criteria**
Age at introduction of CF	Receives solid or semi-solid foods between four and six months of age
SSB consumed	Presence of sugar-sweetened beverages (e.g., industrialized fruit juices, cola, drinkable yogurt, etc.) in infant diet
UP[1] consumed	Presence of ultra-processed foods (industrial formulations made from substances derived from food or synthesized from other organic sources) in infant diet, including but not limited to the following:<br>Industrialized cereals: cookies, pastries, crackers, sliced bread<br>Lunchmeats: ham, sausage<br>Dairy: processed cheese, infant formula, cream<br>Sugars and fats: candy, chocolate, mayonnaise, seasoning<br>Fast foods: hamburger, pizza<br>Baby foods: soup, porridge, puree<br>Others: canned food, industrialized chicken broth, fried food

Supplementary Table 1.

Classification and Components of Infant Feeding Practices in Mexican Infants of the MAS-Lactancia Cohort (2016-2018)

Food diversity[2]	Receives at least four food groups (for infants who receive BF) and at least five groups (for infants who consume milk formula).

CFP classification	Six months	Nine months
Adequate	Adequate BF, absence of SSB and UP foods, and age at introduction of CF more than four months.	Adequate BF, food diversity, absence of SSB and UP foods.
Moderately Adequate	Adequate BF, absence of SSB, age at introduction of CF more than four months, but with consumption of UP foods.	Adequate BF, absence of SSB, but without food diversity and with consumption of UP foods.<br>Adequate BF, absence of SSB and UP foods, but without food diversity.<br>Adequate BF, absence of SSB, with food diversity, but with consumption of UP foods.
Inadequate	Inadequate BF<br>Any type of breastfeeding with the presence of SSB or age at introduction of CF before four months of age.	Inadequate BF<br>Any type of breastfeeding with the presence of SSB.

CFP: Complementary feeding practices, BF: Breastfeeding, CF: Complementary feeding, SSB: Sugar-sweetened beverages; UP: Ultra-processed foods. [1]NOVA system of food classification. [2]The food diversity indicator was considered only at evaluations in the ninth month because at six months infants were initiating CF.

Supplementary Table 2.

Structure of the Questionnaire on Parental Feeding Styles Applied to Participant Mothers

Scales and sub-constructs	Components	Thompson et al. (2009)
Pressuring feeding style		
Pressuring to finish (Parents control feeding due to concern that the child is eating too little)	Behavior Items	PR1 Try to get (child) to finish his/her food<br>PR2 If (child) seems full, encourage to finish anyway<br>PR3 Try to get (child) to finish breastmilk or formula<br>PR4 Try to get (child) to eat even if not hungry<br>PR6 Praise after each bite to encourage finish food
	Belief Items	PR7 Important for toddler finish all food on his/her plate<br>PR8 Important for child finish all milk in his/her bottle
Pressuring to eat cereal (Parents use infant cereal to fill up or calm the child)	Behavior Items	PR11 Give/gave (child) cereal in the bottle
	Belief Items	PR12 Cereal in bottle helps child sleep through the night<br>PR13 Putting cereal in bottle is good b/c it helps infant feel full<br>PR14 An infant <6 mo needs more than formula or breastmilk to be full<br>PR15 An infant <6 mo needs more than formula or breastmilk to sleep through the night
Pressuring to soothe (The parents feed the child to calm him)	Behavior Items	PR16 When (child) cries, immediately feed him/her
	Belief Items	PR17 Best way to make infant stop crying is to feed him/her<br>PR18 Best way to make toddler stop crying is to feed him/her<br>PR19 When infant cries it usually means s/he needs to be fed
Restrictive		
Restrictive in quantity (Parents limits quantities of all foods)	Behavior Items	RS1 I carefully control how much (child) eats<br>RS2 I am very careful not to feed (child) too much
	Belief Items	RS3 Important parent has rules re: how much child eat<br>RS4 Important parent decides how much infant should eat
Restrictive in quality (Parents limit the child's diet to healthy foods)	Behavior Items	RS5 I let (child) eat fast food<br>RS6 I let (child) eat junk food
	Belief Items	RS7 A child should never eat fast food<br>RS8 An infant should never eat fast food

Supplementary Table 2.

Structure of the Questionnaire on Parental Feeding Styles Applied to Participant Mothers

		RS9 A child should never eat sugary food like cookies
		RS10 A child should never eat junk food like chips
Responsive		
Responsive in satiety (The parent is attentive to the child's signals while setting limits)	Behavior Items	RP1 (Child) lets me know when s/he is full
		RP2 (Child) lets me knows when s/he is hungry
		RP3 I let (child) decide how much to eat
		RP4 I pay attention when (child) seems to be telling me that s/he is full or hungry
		RP5 I allow (child) to eat when s/he is hungry
	Belief Items	RP6 Child knows when s/he is full
		RP7 Child knows when hungry, needs to eat
Responsive in attention (Parents encourage exploration in a positive environment)	Behavior Items	RP8 Talk to (child) to encourage to drink formula/breastmilk
		RP9 Talk to (child) to encourage him/her to eat
		RP10 Show (child) how to eat by taking a bite or pretending to
	Belief Items	RP11 I will retry new foods if they are rejected at first
		RP12 Important to help or encourage a toddler to eat

Supplementary Table 3.

Baseline Characteristics of Mothers in the Study Sample versus Mothers Recruited from the MAS- Lactancia Cohort

Continuous variables	Study sample six months		Recruited sample			Study sample nine months		Recruited sample		
	n	M (SD)/%	n	M (SD)/%	p value	n	M(SD)/%	n	M(SD)/%	p value
Age (years)	259	27.0 (5.0)	699	26.3 (5.2)	0.07	234	27.3 (5.0)	738	26.3 (5.2)	0.01
Number of children	259	0.7 (0.9)	686	0.7 (0.9)	0.58	234	0.7 (0.8)	725	0.7 (0.9)	0.71
Formal education (years)	259	16.4 (3.6)	690	16.3 (3.6)	0.68	234	16.7 (3.6)	729	16.2 (3.6)	0.06
Categorical variables										
Employed	259	56.3	691	57.3	0.10	234	57.7	730	56.6	0.28
With couple	258	88.4	695	85.2	0.21	234	83.8	734	85.2	0.13
Household Wealth Index	241	100.0	645	100.0	0.70	218	100.0	681	100.0	0.27
Tertile 1	76	31.5	220	34.5		63	28.9	236	34.7	
Tertile 2	86	35.7	212	32.9		76	34.9	225	33.0	
Tertile 3	79	32.8	213	33.0		79	36.2	220	32.3	

Note: Student t-test was applied and p-values are bolded if significant a $p < 0.05$. Chi-square test or Fisher’s exact test were applied and p-values are bolded if significant a $p < 0.05$.
Sample sizes differ due to missing values in variables

Supplementary Table 4.

Infant Age at Introduction of Complementary Foods by Formula Consumption

	Both		Without formula		With formula		*p*-value
	M (SD)	n	M (SD)	n	M (SD)	n	
Age at introduction of CF	4.28 (2.24)	263	5.79 (0.75)	111	3.17 (2.33)	152	**<0.01**

Note: p-values are bolded if significant a $p < 0.05$.

CF: Complementary feeding. Student *t*-test for mean differences between formula and non-formula categories

Supplementary Table 5.

Longitudinal Association Between Complementary Feeding Practices and Growth, with Parental Feeding Style Sub-Constructs

	Complementary Feeding Practices[2]						Growth[3]		
	Moderately Adequate			Inadequate			Weight-for-length z-score[4]		
PFS sub-constructs[1]	OR	CI 95% (LL,UL)	*p*-value	OR	CI 95% (LL,UL)	*p*-value	β	CI 95% (LL,UL)	*p*-value
Pressuring to finish						0.81‡			0.07‡
Increase†	0.54	(0.09, 03.07)	0.49	0.73	(0.14, 3.81)	0.71	-0.11	(-0.33, 0.11)	0.34
Decrease†	0.97	(0.14, 6.74)	0.97	0.90	(0.14, 5.88)	0.91	-0.28	(-0.50, -0.06)	**0.01**
Pressuring to eat cereal						0.68‡			0.25‡
Increase†	1.23	(0.23, 06.61)	0.81	0.54	(0.11, 2.67)	0.45	0.04	(-0.18, 0.27)	0.70
Decrease†	0.97	(0.13, 07.22)	0.97	0.68	(0.10, 4.68)	0.69	0.11	(-0.11, 0.34)	0.32
Pressuring to soothe						0.74‡			0.26‡
Increase†	2.18	(0.35, 13.66)	0.40	1.66	(0.28, 9.81)	0.58	-0.10	(-0.32, 0.12)	0.37
Decrease†	4.11	(0.37, 45.23)	0.25	3.40	(0.33, 34.85)	0.30	-0.02	(-0.26, 0.22)	0.88
Restrictive in quality						0.74‡			0.29‡
Increase†	1.49	(0.27, 08.10)	0.65	1.08	(0.21, 5.47)	0.93	-0.05	(-0.28, 0.18)	0.66
Decrease†	2.53	(0.38, 16.66)	0.33	2.85	(0.47, 17.25)	0.25	-0.03	(-0.26, 0.19)	0.76
Restrictive in quantity						0.30‡			0.05‡
Increase†	0.26	(0.05, 01.47)	0.13	0.97	(0.20, 4.71)	0.97	-0.21	(-0.43, 0.01)	0.06
Decrease†	2.26	(0.22, 23.79)	0.50	4.88	(0.49, 48.28)	0.18	0.13	(-0.09, 0.35)	0.25
Responsive to satiety						0.54‡			0.26‡
Increase†	0.34	(0.06, 01.93)	0.23	0.54	(0.10, 2.86)	0.47	0.05	(-0.16, 0.25)	0.67
Decrease†	2.67	(0.21, 33.50)	0.45	4.50	(0.38, 53.16)	0.23	0.11	(-0.14, 0.36)	0.40
Responsive in attention						0.80‡			0.23‡
Increase†	0.71	(0.12, 04.37)	0.71	0.75	(0.13, 4.24)	0.74	-0.10	(-0.32, 0.12)	0.37
Decrease†	0.56	(0.09, 03.59)	0.54	0.43	(0.07, 2.63)	0.36	-0.12	(-0.35, 0.12)	0.32

Note: *p*-values are bolded if significant a $p < 0.05$.

[1]Assessment of the seven parental feeding style sub-constructs corresponding to pressuring, restrictive and responsive feeding styles. [2]Multinomial logistic regression models adjusted by morbidity, birthweight, type of caregiver, BMI Z-score, educational background, number of children, BMI at recruitment and

Supplementary Table 5.

Longitudinal Association Between Complementary Feeding Practices and Growth, with Parental Feeding Style Sub-Constructs

mother's Household Wealth Index (HWI) tertile, with adequate complementary feeding practices as the reference category [3] Lineal regression models adjusted by the same covariates mentioned previously, except by BMI Z-scores, and the addition of "type of breastfeeding",[4] Differences in weight-for-length Z-score between nine and six months. PFS: Parental feeding styles, OR: Odds ratio, CI: Confident intervals; LL: Lower limit; UL: Upper limit., [†]Versus without change between six and nine months as reference category in regression model, [‡]*p*-value of the global model.
